# Differences in the temporal patterns of occupational time on feet and sitting between homecare and nursing home workers

**DOI:** 10.1093/annweh/wxaf049

**Published:** 2025-08-29

**Authors:** Nestor Lögdal, Svend Erik Mathiassen, Jennie A Jackson, David M Hallman

**Affiliations:** Department of Occupational Health, Psychology, and Sports Sciences, University of Gavle, Kungsbäcksvägen 47, 801 76 Gävle, Sweden; Department of Occupational Health, Psychology, and Sports Sciences, University of Gavle, Kungsbäcksvägen 47, 801 76 Gävle, Sweden; Department of Occupational Health, Psychology, and Sports Sciences, University of Gavle, Kungsbäcksvägen 47, 801 76 Gävle, Sweden; Department of Occupational Health, Psychology, and Sports Sciences, University of Gavle, Kungsbäcksvägen 47, 801 76 Gävle, Sweden

**Keywords:** occupational physical activity, physical behaviors, physical demands

## Abstract

**Background:**

Eldercare work is physically demanding, which may contribute to high rates of sickness absence. Understanding the temporal patterns of time on feet and sitting, the latter assumed to represent recovery, and how they depend on organizational and individual factors is key to a better work organization that can effectively promote health, but this has not been studied before.

**Aim:**

To describe temporal patterns of time on feet and sitting among homecare and nursing home workers and examine their associations with setting, job demands and resources, job title, and age.

**Methods:**

Swedish homecare (*n* = 101) and nursing home (*n* = 73) workers wore accelerometers for 7 consecutive days to assess physical behaviors. Short (≤5 min), moderate (>5 to ≤30 min), and long (>30 min) bouts of time on feet and sitting were identified, with their relative distribution expressed in 5 ratios and analyzed using compositional data analysis procedures. Workers also completed a questionnaire on job organizational factors (job demands and resources) and individual factors (job title and age). MAN(C)OVA models were used to analyze differences in behaviors between eldercare settings, with organizational and individual factors being added in 2 consecutive models. Univariate analyses followed the multivariate models.

**Results:**

Homecare and nursing home workers spent most of the workday on their feet (51.9% and 56.9%, respectively). The only statistically significant difference between settings was that homecare workers spent 30.1% less time sitting in long bouts relative to moderate and short compared to nursing home workers (*P* = 0.011), and this difference remained after adding covariates. Higher age was associated with more time on feet relative to sitting (*P* = 0.002, η^2^ = 0.06) and more time on feet in long bouts relative to moderate and short bouts (*P* = 0.001, η^2^ = 0.06) with medium effect sizes, as well as to more time on feet in moderate bouts relative short bouts (*P* = 0.011, η^2^ = 0.04), and less sitting in long bouts relative to moderate and short bouts (*P* = 0.019, η^2^ = 0.03) with small effect sizes.

**Conclusions:**

Temporal patterns varied by setting and age, with homecare workers sitting less in long bouts than nursing home workers, and older workers spending more time on feet than younger workers. These findings suggest that work setting and worker characteristics are associated with temporal patterns of physical behavior, although most effects were small-to-moderate and their practical relevance remain uncertain and warrants further study.

**What’s Important About This Paper?**

Eldercare workers experience high rates of pain and sickness absence, linked to physically demanding work. This study examined temporal patterns of time on feet and sitting, comparing homecare and nursing home workers. While both groups spent most of their workday on their feet, they accumulated this time differently. These findings are important for organizing work in ways that allow for physical behaviors that support health.

## Introduction

Eldercare workers face high rates of musculoskeletal pain and sickness absence (OECD 2020; Swedish National Board of Social Affairs and Health 2025) both of which have been associated with higher occupational physical demands and perceived exertion (Andersen et al. 2012a, 2012b, 2013). Efforts to decrease physical demands have largely focused on reducing exposure to physically demanding tasks, eg lifts and transfers, by introducing aids such as assistive devices (Owen and Garg 1991; Garg and Owen 1992, 1994; Owen et al. 1992; Hsieh et al. 2022). While the use of aids has been successful in reducing perceived exertion during task execution (Garg and Owen 1992, 1994; Owen and Garg 1994; Owen and Fragala 1999), perceived exertion across the entire workday (Karstad et al. 2018; Januario et al. 2019, 2020) and sickness absence rates (OECD 2020; Swedish National Board of Social Affairs and Health 2025) remain high, indicating that other occupational physical demands may contribute to these outcomes.

Time on feet, including standing and walking, is a physical behavior that has received limited attention in research on eldercare workers. The few existing studies indicate that workers spend half to two-thirds of their workday on their feet (Januario et al. 2020; Tjøsvoll et al. 2022) and that they rate time on feet as physically demanding (Yeung 2012). In other occupations, prolonged time on feet has been linked to adverse health effects, including cardiovascular disease, musculoskeletal fatigue, pain, and discomfort (Waters and Dick 2015; Coenen et al. 2017, 2018; Øverås et al. 2020; European Agency for Safety and Health at Work 2021) as well as to increased sickness absence (Gupta et al. 2020a). The temporal pattern of time on feet and sitting—measured through the duration and distribution of uninterrupted bouts—has also been shown to influence health (Waters and Dick 2015; European Agency for Safety and Health at Work 2021). However, the temporal patterns of time on feet and sitting have not been studied in eldercare workers.

Eldercare includes both nursing homes, where care is provided in specialized facilities, and homecare, where caregivers assist individuals in their own homes (Szebehely et al. 2017). Differences in work organization and tasks between settings may influence the temporal patterns of physical behaviors. Nursing home workers typically stay in a single location (Schön et al. 2016; Swedish Association of Local Authorities and Regions 2020) and work in close proximity to colleagues. This allows for quick assistance and coordination of tasks, and it may offer workers more control over how their work is organized, potentially enabling them to schedule tasks in ways that create longer, uninterrupted periods for sitting. In contrast, homecare workers typically work alone (Wipfli et al. 2012) and provide care in multiple client homes, performing all necessary tasks at each site before traveling to the next. In Scandinavia, this travel occurs by car, bike, or walking, depending on factors such as urban or rural location, seasonal conditions, and time of day (Cœugnet et al. 2016; Helgheim and Sandbaek 2021; Liaset et al. 2023, 2024a; Norström et al. 2025). Many homecare organizations use time-scheduling systems that impose strict time frames for in-home visits and transportation (Holm and Angelsen 2014; Burns et al. 2023). These systems are essentially creating a homecare analogue of Methods-Time Measurement systems used in assembly work, where tasks are broken down into standardized basic motions and assigned fixed time values to optimize workflow and minimize variability (Dempsey et al. 2010). Frequent travel and short time windows at each worksite during which tasks must be performed may limit opportunities for homecare workers to get longer continuous bouts of sitting. Additionally, traveling between homes often involves time on feet, such as walking to and from a car, further contributing to differences in physical behavior patterns between nursing homes and homecare. Considering potential differences in temporal patterns of time on feet and sitting is key to understanding the unique demands of each care setting and designing targeted interventions for sustainable physical behaviors.

Other organizational factors, like job demands and resources, may also differ between homecare and nursing home settings, influencing temporal patterns of time on feet and sitting (Januario et al. 2020). Research on nursing home workers has yielded mixed findings regarding job demands, resources, and temporal physical behaviors, with 1 study reporting no association between quantitative demands, influence at work, and hourly step rate (Stevens et al. 2021), and another finding marginal associations between influence at work and time on feet (Januario et al. 2020). A scoping review of nursing in eldercare and hospital settings found that high job demands, low supervisor support, and limited control of work schedules reduced opportunities for workers to take breaks, for instance represented by periods of sitting, while supportive leadership and clear break policies facilitated breaks (Wendsche et al. 2017). Manageable demands and sufficient control over tasks may allow workers to incorporate breaks to sit down and rest, while high demands, time pressure, and limited control may lead to prolonged time on feet and reduced sitting.

Individual factors such as education and age may also affect the temporal pattern of time on feet and sitting (Stevens et al. 2021). In both homecare and nursing homes, worker’s education levels are related to job titles, responsibilities, and hence tasks (Engström et al. 2011; Laxer et al. 2016). For example, nurse’s aides primarily perform basic care tasks, while assistant nurses, who typically have formal training, may have additional responsibilities requiring increased technical or administrative skills, such as seated data entry. Thus, education can determine the tasks in the job, and thus the physical behaviors (Laxer et al. 2016). Similarly, age may influence physical behaviors (Althoff et al. 2017). Older care workers report greater difficulties with physically demanding tasks and prolonged periods on feet than younger workers (Fragar and Depczynski 2011), suggesting that older workers may actively seek less physically demanding and/or seated tasks. This could lead to age-related differences in tasks performed and thus to differences in the temporal patterns of time on feet and sitting.

This cross-sectional study aimed to describe temporal patterns of time on feet and sitting among homecare and nursing home workers and examine their associations with setting, job demands and resources, job title, and age. We hypothesized that homecare workers would spend more time on feet relative to sitting compared to nursing home workers and that time on feet would mainly occur in short and moderate bouts. We hypothesized that higher job demands would be associated with increased time spent on feet relative to sitting. Conversely, we hypothesized that higher levels of influence would be associated with decreased time on feet relative to sitting. We hypothesized that assistant nurses would spend less time on feet relative to sitting compared to nurse’s aides. Finally, we hypothesized that higher age would be associated with less time on feet.

## Methods

### Study design and population

Data for the study were collected between June 2021 and June 2024 as part of a larger project collecting organizational and individual data on the psychosocial and physical work environment, workplace attitudes, and health using questionnaires, accelerometers, diaries, interviews, and registers (Svensson et al. 2022). We invited 1,380 eldercare workers from 27 homecare units and 22 nursing home units (in 12 nursing homes) in 3 different municipalities in Sweden to participate in accelerometer measurements. The municipalities are located in the south, middle, and northern parts of Sweden and vary in size, with populations ranging from 32,000 to 103,000 and include both rural and urban areas and both homecare and nursing home services. Participants were recruited via email and at monthly staff meetings. During the staff meetings, a member of the research team presented the research project and invited staff to participate in the accelerometer and/or questionnaire data collections. The workers received either a paper version of the questionnaire to fill out during the meetings or received a link via email to a digital version of the questionnaire. All participants provided their written informed consent. The study was approved by the Swedish Ethical Review Authority (Ref. no. 2019-06220).

### Accelerometer measurements

Workers agreeing to participate in the accelerometer measurements met individually with a member of the research team to go through the data collection protocol. A triaxial accelerometer (Axivity AX3, Axivity Ltd, Newcastle, UK) was then placed on the front of the right thigh and secured with waterproof medical tape. The accelerometer had a sampling frequency of 25 Hz and a range of ±8 G and was worn around the clock for 7 consecutive days. Additionally, each worker was asked to record the time at which they started and stopped work and when they went to bed and got up in a provided paper diary. Each participant’s height and mass were measured when they wore light clothes (eg t-shirt and pants) and no shoes. Height was measured to the nearest 0.5 cm using a wall mounted measuring tape, and body mass to the nearest 0.1 kg on a portable, digital scale (Beurer GmbH, BF183). From the anthropometric measurements, body mass index (BMI) was calculated by dividing body mass by the height squared (kg/m^2^).

### Data processing

The accelerometer data were processed using ActiPass (Hettiarachchi and Johansson 2023), a validated custom-built software in the MATLAB environment (Skotte et al. 2014). The software classifies physical behaviors into the following mutually exclusive categories: *Lying down*, *Sitting*, *Standing still*, *Standing with* s*mall movements, Walking*, *Running*, *Stair*-*climbing*, and *Cycling.* Two mutually exclusive behaviors were derived from the ActiPass analysis during working hours: *On feet* encompassing time spent *Standing still*, *Small movements while standing, Walking*, *Running*, *Stair*-*climbing*, and *Cycling,* and *Sitting* including time spent *sitting* and *lying down* (negligible during work hours).

#### Exposure variation analysis.

The temporal patterns of time on feet and sitting were both quantified using exposure variation analysis (EVA; Mathiassen and Winkel 1991; Mathiassen 2006; Hallman et al. 2015). A default EVA matrix was calculated by the ActiPass software in which uninterrupted bouts were determined for both time on feet and sitting, in bins with durations of: <1, 1 to 2, 2 to 3, 3 to 4, 4 to 5, 5 to 10, 10 to 30, 30 to 60, and >60 min. We merged the default EVA categories into “short,” “moderate,” and “long” bouts (≤5 min, 5 to 30 min, and >30 min, respectively) of time on feet and sitting, expressed as percentages of total work time. The merged bout categories were determined based on a trade-off between providing sufficient physiologically relevant detail in the observations, minimizing the number of zeros in the data (which cannot be handled in compositional data analyses, as discussed below), and previous research on blue-collar workers (Gupta et al. 2016).

#### Compositional data analysis.

Over a set period, the times spent in the different physical behaviors will together sum to the total period time. Such data are compositional, meaning that changes in the time spent in 1 behavior category will affect the time spent in other behaviors. Therefore, the data in our study were processed using compositional data analysis (CoDA) (Dumuid et al. 2020; Gupta et al. 2020b). One limitation of CoDA procedures is that they do not allow for zero time in any of the included categories. To address this, the data set was inspected for zeros in all participants. For participants with a single zero-containing cell, 0.5% was re-allocated to that cell by subtracting 0.1% from each of the 5 other cells. For participants with 2 zero-containing cells, 0.5% was re-allocated to both zero-containing cells by subtracting 0.25% from each of the remaining 4 cells. No more than 2 zero-containing cells were found for any of the participants. A total of 69 zero-containing cells (in 57 individuals) were identified among the 2,088 total cells, all of which occurred in the long bout categories, either on feet or sitting. The compositions were then transformed into 5 orthogonal isometric log ratio (ILR) coordinates allowing for further analysis using conventional statistical methods (Dumuid et al. 2018):


$$\begin{array}{l} ILR\;1\;=\;\sqrt{\frac{9}{6}}\;*ln\left(\frac{\sqrt[{3}]{Time\;on\;feet\;in\;long\;bouts\;*\;Time\;on\;feet\;in\;moderate\;bouts\;*\;Time\;on\;feet\;in\;short\;bouts}}{\sqrt[{3}]{Time\;sitting\;in\;long\;bouts\;*\;Time\;sitting\;in\;moderate\;bouts\;*\;Time\;sitting\;in\;short\;bouts}}\right) \end{array}$$ .

$$\begin{array}{l} ILR\;2\;=\;\sqrt{\frac{2}{3}}\;*ln\left(\frac{Time\;on\;feet\;in\;long\;bouts}{\sqrt[{2}]{Time\;on\;feet\;in\;moderate\;bouts\;*\;Time\;on\;feet\;in\;short\;bouts}}\right) \end{array}$$

$$\begin{array}{l} ILR\;3\;=\;\sqrt{\frac{1}{2}}\;*ln\left(\frac{Time\;on\;feet\;in\;moderate\;bouts}{Time\;on\;feet\;in\;short\;bouts}\right) \end{array}$$

$$\begin{array}{l} ILR\;4\;=\;\sqrt{\frac{2}{3}}*\ln\left(\frac{Time\;sitting\;in\;long\;bouts}{\sqrt[{2}]{Time\;sitting\;in\;moderate\;bouts\;*\;Time\;sitting\;in\;short\;bouts}}\right) \end{array}$$

$$\begin{array}{l} ILR\;5\;=\;\sqrt{\frac{1}{2}}*\ln\left(\frac{Time\;sitting\;in\;moderate\;bouts}{Time\;sitting\;in\;short\;bouts}\right) \end{array}$$

ILR 1 represents total time on feet relative to total time sitting, while the other 4 ILRs represent orthogonal ratios between short, moderate, and long bouts on feet and in sitting.

#### Organizational and individual factors.

Job demands were assessed using the quantitative demands dimension (3 items, QD1, QD2, QD3, eg *How often do you not have time to complete all your work tasks?*) and resources were assessed using the influence at work dimension (4 items, INX1, IN2, IN3, IN4, eg *Can you influence the amount of work assigned to you?*), all 7 items from the third Copenhagen Psychosocial Questionnaire (COPSOQ III) (Berthelsen et al. 2020). The response scales had 5 response options labeled: *Always*, *Often*, *Sometimes*, *Seldom*, *Never/hardly ever*, with the scale range 0 to 100. Indices were computed as each participant’s mean score across the individual items. For an index to be computed, the respondent had to have answered at least half of the items. Age, gender, percent of fulltime work, and job title were collected from the organizations. Age was assessed through year of birth, and job title was categorized into 2 levels: assistant nurse and nurse’s aide.

#### Eligibility criteria.

For this study we included data only from permanently employed assistant nurses and nurse’s aides who were working day and/or evening shifts (not night) and were involved in direct care work and accelerometer data only from working hours. Further, to be included, workers were required to have at least 1 workday with ≥4 h of accelerometer recordings and to have completed questionnaire responses for our demand- and resource variables.

### Statistical analyses

The data were analyzed in R (R Core Team 2023) using the tidyverse suite of packages (Wickham et al. 2019). Data are presented as means and standard deviations (SD) or counts and proportions. The dependent variables showed no multicollinearity, heteroscedasticity, or residuals with pronounced deviations from normal distributions. We used partial eta squared (η_p_^2^) as a standardized effect size measure with small (0.01), medium (0.06), and large (0.14) effect sizes defined according to Cohens recommendations (Cohen 1988). The alpha level was set to *P* < 0.05.

#### Comparison of physical behaviors between settings.

We analyzed the data in 3 different models, 1 multivariate analysis of variance (MANOVA), and 2 multivariate analyses of covariance (MANCOVA) using setting as the independent variable and the 5 ILRs as dependent variables.

Model 1 tested differences in the temporal patterns of physical behaviors between homecare and nursing home workers without covariates. In Model 2, factors at the organizational level were considered in addition to the main effect of setting by adding quantitative demands and influence at work indices as covariates, and in Model 3 factors at the individual level were considered in addition to the main effects of setting and factors at the organizational level by adding job title and age as covariates. The multivariate models were followed up by univariate analyses of variance (ANOVA) with covariates added (ANCOVA) in the same 3-step approach as in the multivariate models, first analyzing setting differences alone, then incorporating organizational-level factors, and finally including even individual-level factors.

## Results

### Demographic data

In total, 174 workers (101 in homecare and 73 in nursing homes) had sufficient accelerometer and questionnaire data to be included in the study. On average, homecare workers were younger than nursing home workers, comprised a lower proportion of men and foreign-born workers, and had worked fewer years in eldercare, while BMI, percent of fulltime work, and duration of the accelerometer recording were similar between the groups (Table 1).

**Table 1.** Demographic data for homecare and nursing home workers

Variable	Eldercare setting	
	Homecare (*n* = 101)	Nursing home (*n* = 73)
Women, *n* (%)	83 (82.2)	71 (97.3)
Age (years), mean (SD)	48.7 (11.2)	50.0 (10.4)
Body mass index (kg/m^2)^, mean (SD)	28.7 (5.7)	28.2 (5.8)
Born in Sweden, *n* (%)		
Yes	73 (72.3)	46 (63.0)
No	27 (26.7)	24 (32.9)
Missing	1 (1)	3 (4.1)
Marital status, *n* (%)		
Married/registered partner/cohabitant	62 (61.4)	49 (67.1)
Single household	39 (38.6)	21 (28.8)
Missing	0 (0.0)	3 (4.1)
Years worked in current setting, mean (SD)	15.5 (12.2)	19.2 (13.9)
Percent of fulltime work, mean (SD)	93.0 (14.0)	94.8 (9.4)
Job title, *n* (%)		
Assistant nurse	100 (78.7)	79 (88.8)
Nurse’s aide	27 (21.3)	10 (11.2)
Average number of measurement days per participant, mean (SD)	4.7 (1.7)	4.5 (1.4)
Average number of measurement hours per participant, mean (SD)	34.2 (15.7)	33.1 (11.8)
Quantitative demands (0 to 100), mean (SD)	41.2 (19.5)	44.7 (16.2)
Influence at work (0 to 100), mean (SD)	37.5 (20.1)	37.5 (18.8)

### Comparison of physical behaviors between settings

**Fig. 1.**
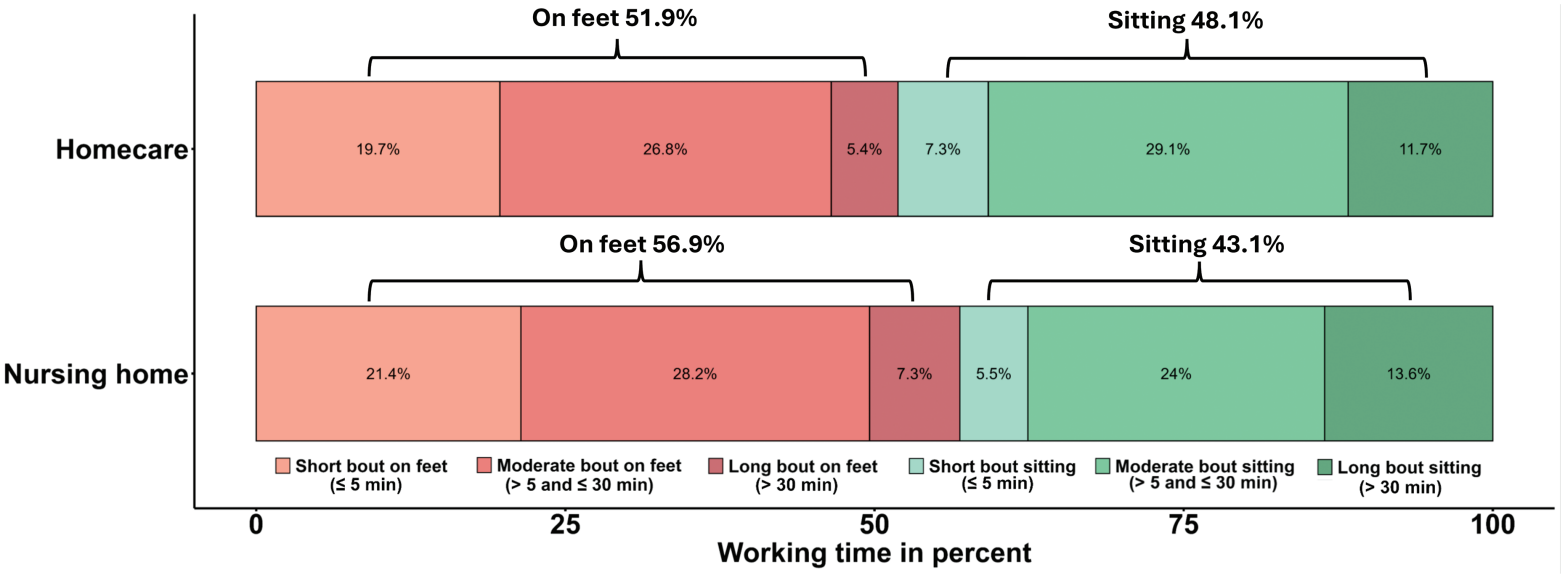
Average percentage of time spent on feet and in sitting in the 3 investigated bout durations for homecare and nursing home workers.

**Fig. 2.**
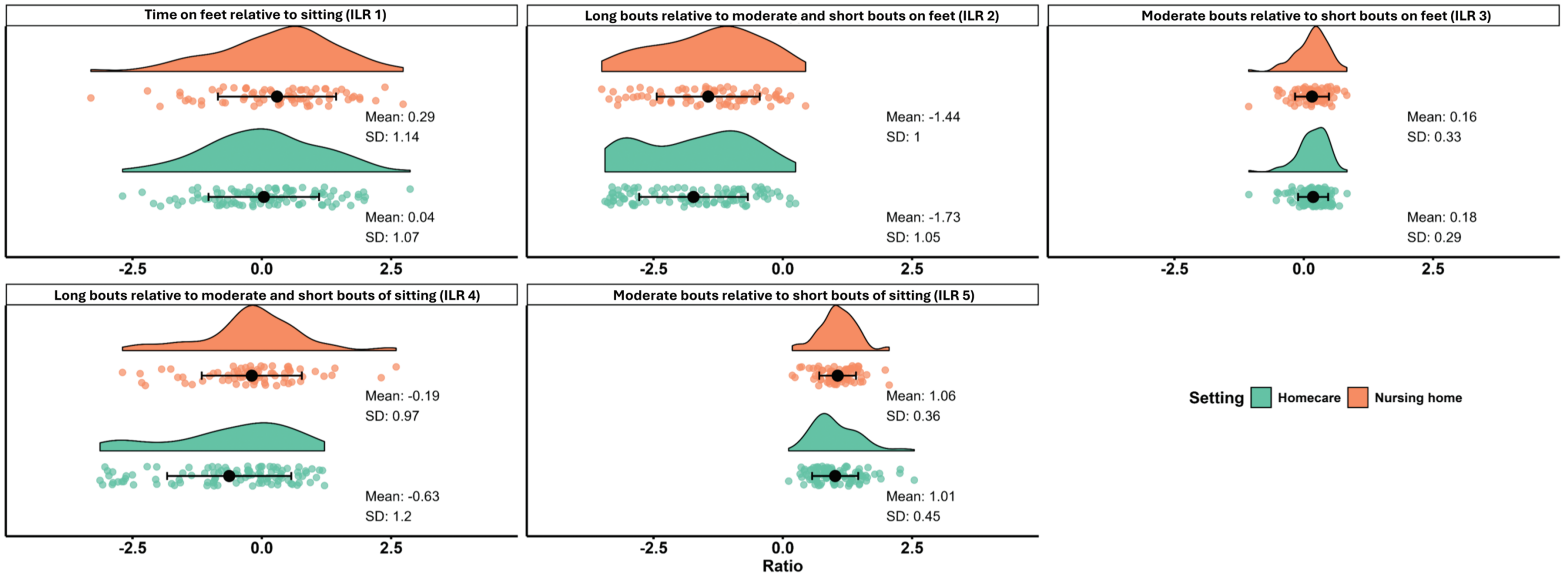
Mean (black dot) and standard deviation (black horizontal lines) as well as individual data points (colored dots) and their distribution (colored density curves) for the isometric log ratios (ILRs) among homecare (green) and nursing home workers (orange). Positive ILR values indicate more time spent in the physical behavior(s) listed before the word “relative.” For example, positive values for time spent on feet relative to sitting (ILR 1, top left) means more time on feet than in sitting.

The average proportion of working time spent in each of the 6 behavior categories for both home care and nursing home workers are presented in Fig. 1. Both groups spent most of the working day on feet, with homecare workers at 51.9% and nursing home workers at 56.9% working time (Fig. 1). Fig. 2 shows the mean and SD of the 5 ILRs, along with individual data points and distributions for homecare workers (green) and nursing home workers (orange).

In Model 1, the MANOVA showed a statistically significant difference between eldercare settings and the effect size was large (Table 2). The difference between settings remained in both Model 2 when organizational-level factors were added as covariates, and in Model 3 with both organizational- and individual-level factors added (Table 2). The subsequent univariate analyses showed a statically significant difference between settings for ILR4 (Table 3, Models 1 to 3), with homecare workers spending less time sitting in long bouts relative to moderate and short bouts compared to nursing home workers (Fig. 2).

**Table 2.** Results from the multivariate analyses.

	Model 1				Model 2				Model 3			
Factor	DF	*F*	*P*	η_p_²	DF	*F*	*P*	η_p_²	DF	*F*	*P*	η_p_²
Eldercare setting	1, 172	5.38	**<0.001**	**0.14**	1, 170	5.35	**<0.001**	**0.14**	1, 168	5.32	**<0.001**	**0.14**
Quantitative demands	-	-	-	-	1, 170	1.30	0.265	0.04	1, 168	1.39	0.230	0.04
Influence at work	-	-	-	-	1, 170	1.45	0.210	0.04	1, 168	1.51	0.189	0.04
Job title	-	-	-	-	-	-	-	-	1, 168	1.10	0.364	0.03
Age	-	-	-	-	-	-	-	-	1, 168	4.01	**0.002**	**0.11**

Statistically significant *P*-values and effect sizes are marked in bold text.

**Table 3.** Results from the univariate analyses.

	Time on feet relative to time sitting (ILR 1)				Long bouts relative to moderate and short bouts on feet (ILR 2)				Moderate bouts relative to short bouts on feet (ILR 3)				Long bouts relative to moderate and short bouts of sitting (ILR 4)					Moderate bouts relative to short bouts of sitting (ILR 5)				
	DF	*F*	*P*	η^2^	DF	*F*	*P*	η^2^	DF	*F*	*P*	η^2^		DF	*F*	*P*	η^2^		DF	*F*	*P*	η^2^
Model 1																						
Eldercare setting	1, 172	2.28	0.133	0.01	1, 172	3.26	0.073	0.02	1, 172	0.21	0.646	0.00		1, 172	6.54	**0.011**	**0.04**		1, 172	0.59	0.445	0.00
Model 2																						
Eldercare setting	1, 170	2.29	0.132	0.01	1, 170	3.31	0.071	0.02	1, 170	0.22	0.642	0.00		1, 170	6.47	**0.012**	**0.04**		1, 170	0.58	0.447	0.00
Quantitative demands	1, 170	0.37	0.544	0.00	1, 170	3.25	0.073	0.02	1, 170	0.46	0.499	0.00		1, 170	0.18	0.671	0.00		1, 170	0.76	0.383	0.00
Influence at work	1, 170	2.46	0.119	0.01	1, 170	1.01	0.316	0.01	1, 170	6.26	**0.013**	**0.04**		1, 170	0.09	0.759	0.00		1, 170	0.15	0.699	0.00
Model 3																						
Eldercare setting	1, 168	2.40	0.123	0.01	1, 168	3.49	0.064	0.02	1, 168	0.23	0.633	0.00		1, 168	6.62	**0.011**	**0.04**		1, 168	0.58	0.449	0.00
Quantitative demands	1, 168	0.39	0.534	0.00	1, 168	3.43	0.066	0.02	1, 168	0.48	0.488	0.00		1, 168	0.18	0.668	0.00		1, 168	0.76	0.386	0.00
Influence at work	1, 168	2.59	0.110	0.01	1, 168	1.07	0.303	0.01	1, 168	6.60	**0.011**	**0.04**		1, 168	0.10	0.757	0.00		1, 168	0.15	0.700	0.00
Job title	1, 168	0.64	0.425	0.00	1, 168	0.05	0.829	0.00	1, 168	4.61	**0.033**	**0.03**		1, 168	0.04	0.839	0.00		1, 168	0.12	0.727	0.00
Age	1, 168	9.97	**0.002**	**0.06**	1, 168	11.22	**0.001**	**0.06**	1, 168	6.64	**0.011**	**0.04**		1, 168	5.64	**0.019**	**0.03**		1, 168	0.19	0.664	0.00

Statistically significant *P*-values and effect sizes are marked in bold text.

### Effects of additional organizational and individual factors on physical behaviors

In Model 2, the MANCOVA with organizational-level factors added as covariates showed no statistically significant associations between quantitative demands or influence at work and physical behavior (Table 2). However, the univariate analyses (Table 3) indicated a statistically significant association between influence at work and time on feet spent in moderate bouts relative to short bouts (Models 2 and 3, ILR 3), with higher perceived influence being associated with less time on feet in moderate bouts relative to short (Supplementary Fig. S2, ILR 3).

In Model 3, the MANCOVA with both organizational- and individual-level factors showed no statistically significant association between job title and the physical behavior, while a significant association were found for age with a large effect size (Table 2, Model 3). Univariate analysis showed a statistically significant association between job title and time spent in moderate bouts on feet relative to short bouts (Table 3, Model 3, ILR 3), with assistant nurses spending less time in moderate bouts relative to short bouts on feet than nurse’s aides (Supplementary Fig. S3, ILR 3). The univariate analyses also showed statistically significant associations between age and several physical behaviors (Table 3, Model 3, ILR 1 to 4). Higher age was associated with more time on feet relative to sitting, less time sitting in long bouts relative to moderate and short bouts, less time in long bouts on feet relative to moderate and short bouts, and less time in moderate bouts on feet relative to short bouts (Supplementary Fig. S4, ILR 1-4).

## Discussion

The aim of this cross-sectional study was to describe temporal patterns of time on feet and sitting among homecare and nursing home workers and examine their associations with setting, job demands and resources, job title, and age. To our knowledge, this is the first study to compare occupational physical behaviors between homecare and nursing home workers using a CoDA framework. It adds to the limited body of research that uses reliable technical measurements to assess physical exposure in eldercare, by examining temporal patterns of both standing/moving and sitting behaviors across full workdays. By applying CoDA, the study appropriately accounts for the codependency inherent in time-use data. The study also employs a multilevel analytic strategy across the 3 models, thereby adjusting for both organizational (eg quantitative demands, influence) and individual (eg age, job title) factors. Moreover, it is the first study to explore age-related differences in these compositional temporal patterns among eldercare workers using accelerometry and stratified ILRs. This multidimensional approach provides a more nuanced understanding of how eldercare setting and worker characteristics jointly influence occupational physical behavior.

We found that eldercare workers spent proportionally more time in total on feet relative to sitting in both nursing homes and home care settings, but that the temporal patterns of behavior differed between settings, with homecare workers spending less time in longer bouts of sitting relative to shorter bouts (indicating more frequent interruptions) than nursing home workers. Job demands were not associated with any of the behaviors. Nurse’s aides and those reporting less influence at work accumulated more of their working hours on feet, and in longer bouts (ie fewer interruptions). Higher age was associated with more time on feet relative to sitting and more time in shorter bouts in both behaviors (ie more interruptions). These results can guide future research and workplace initiatives in the eldercare sector, as well as inspire workplace interventions supporting sustainable work practices by ensuring adequate opportunities for recovery throughout the workday.

We expected homecare workers to spend more time on feet than nursing home workers due to travel requirements and differences in work organization, such as time-scheduling systems (Holm and Angelsen 2014; Cœugnet et al. 2016; Burns et al. 2023). However, the only significant difference observed was that homecare workers spent approximately 30% less time in long bouts of sitting (>30 min) relative to moderate and short bouts than nursing home workers. One possible explanation is that in homecare, work is divided into shorter periods by travel between clients. While this travel often involves sitting (eg driving), it typically lasts less than 30 min and may limit opportunities for longer uninterrupted bouts of sitting during the workday (Cœugnet et al. 2016; Andersson and Sjölund 2023). The larger variation in physical behavior patterns among homecare workers may benefit musculoskeletal health by introducing more variation, but having insufficient opportunities for continuous sitting may also reduce chances to recover during work (Waters and Dick 2015; Buffey et al. 2022). Moreover, qualitative studies suggest that homecare workers experience travel as a major stressor due to tight schedules, traffic congestion, and parking difficulties (Grasmo et al. 2021; Liaset et al. 2024). Travel time is also often unaccounted for in time-scheduling systems (Strandell 2023), further adding to perceived stress. Nursing home workers, on the other hand, may have greater control over their work structure, allowing them to schedule tasks such as documentation (eg charting) into longer bouts of uninterrupted sitting. Additionally, since nursing home residents generally have higher care needs (Swedish Association of Local Authorities and Regions 2017), tasks like assisting with meals may contribute to extended sitting periods. Differences in break opportunities could also play a role; nursing home workers typically have access to designated break areas, eg when eating, while homecare workers may have to eat on the go or return to their unit between visits. These explanations remain speculative, and further research is needed to clarify the underlying reasons for the behavioral differences and whether sitting time in homecare and nursing home settings provides equivalent recovery opportunities. Other observed differences in physical behavior patterns were not statistically significant, and these interpretations are speculative and should be investigated in future studies.

On average, homecare (51.9%) and nursing home workers (56.9%) spent similar proportions of their workday on their feet. These findings align with results for Norwegian homecare workers (Tjøsvoll et al. 2022), possibly reflecting similarities in work organization or care models. In contrast, higher values of time on feet have been reported for Danish nursing home workers (Januario et al. 2020) maybe due to differences in job tasks, staffing levels, or methodological factors between studies. Spending extended time on feet (eg >30 min at a time or >4 h total per day), whether standing or actively moving, has been associated with various negative health outcomes (Waters and Dick 2015; Øverås et al. 2020; European Agency for Safety and Health at Work 2021; Janssen and Voelcker-Rehage 2023; Ahmadi et al. 2024). Systematic reviews of observational studies have associated prolonged standing (Coenen et al. 2018) and extensive time on feet (Øverås et al. 2020) with increased risks of musculoskeletal discomfort and pain, while increasing the time sitting is associated with less musculoskeletal discomfort and pain. A review of controlled laboratory studies found a dose–response relationship between time in continuous standing and clinically relevant pain, leading to recommendations to limit periods of uninterrupted standing to less than 40 min (Coenen et al. 2017). Additionally, a recent analysis of UK Biobank data found an increased risk of orthostatic circulatory disease associated with more than 2 h of total standing per day (Ahmadi et al. 2024).

In the current study, both homecare and nursing home workers were, on average, well above 2 h of time on feet, which may contribute to an increased risk of adverse health outcomes. However, our data also suggested that uninterrupted bouts on feet were typically shorter than 40 min, and that less than 10% of the worktime was spent in long bouts on feet (>30 min). However, time on feet, and, thus, time spent sitting varied considerably between individuals in both settings, with some workers having far less opportunities for seated recovery than the average, suggesting that health risks were likely unevenly distributed in both groups. However, given the cross-sectional nature of this study, no causal conclusions can be drawn about these associations, and future longitudinal research is needed to clarify whether specific behavioral patterns during work contribute to long-term health outcomes.

Our multivariate models did not find any significant associations between job demands or resources and the temporal patterns of time spent on feet or sitting. However, the univariate analyses (Model 2 and Model 3) found associations between more influence and less time on feet in moderate bouts relative to short bouts, indicating that workers perceiving higher influence had more frequent opportunities to sit down. This result should, however, be interpreted with caution, since the multivariate models did not indicate any effect of influence. Previous studies in unlicensed nursing home workers have shown mixed results regarding associations between job demands, resources, and physical behaviors (Januario et al. 2020; Stevens et al. 2021) while a review of registered nurses working in hospital and eldercare settings found that high job demands, limited supervisor support, and restricted control over work schedules were associated with taking fewer breaks, while supportive leadership and clear break policies promoted taking breaks (Wendsche et al. 2017).

These results suggest that psychosocial factors in the work environment, including influence, may play a role in how individual workers structure their time on feet and in sitting, potentially by influencing the organization of their work tasks or breaks. Interventions to enhance worker’s control over tasks and breaks may lead to better management of physical behaviors for the individual worker, although further research is needed to explore these possibilities.

We did not find any associations between job title and physical behaviors in our multivariate model, possibly reflecting that differences in education and responsibilities do not lead to any major effects on the tasks performed (Engström et al. 2011; Laxer et al. 2016). Nevertheless, the univariate analyses (Table 3, Model 3, ILR 3) showed associations between job title and time on feet in moderate bouts relative short bouts, with assistant nurse’s spending less time on feet in moderate bouts relative to short bouts than nurse’s aides (Supplementary Fig. S3, ILR 3).

We hypothesized that higher age would be associated with less time on feet (lower workload); however, we observed the opposite, where higher age was associated with (i) more time on feet relative to sitting (ILR 1), (ii) more time in long bouts relative to moderate and short bouts on feet (ILR 2), and (iii) more time in moderate bouts on feet relative to short (ILR 3). Higher age was also associated with less time sitting in long bouts relative to moderate and short bouts (ILR 4). Previous research has found mixed results. For instance, Merkus et al. (2019) found that older healthcare workers had similar or even greater physical workload exposure than younger workers, and thus older workers had higher relative cardiovascular strain while also having reduced aerobic capacity, while Jacobsen et al. (2022) found no association between age and workload. In office settings, Keown et al. (2018) found that older workers had more time on feet compared to younger workers, while Johansson et al. (2020) found that older workers in office settings moved less compared to younger workers.

These mixed findings could reflect how individual older workers adapt their work roles and environments over time. Some may shift toward less physically demanding tasks, such as administrative or specialist functions, or avoid settings with unpredictable workflows and limited collegial support, such as wards with variable staffing (Durosaiye et al. 2016). Others may remain in physically active roles but adopt selective strategies, such as self-pacing, adjusting task boundaries, or informally specializing in duties that draw on experience rather than physical effort (Müller et al. 2013; Mc Carthy et al. 2018). These interpretations remain speculative, and further research is needed to understand the underlying mechanisms.

A key strength of this study is the use of technical measurements to assess time spent on feet and sitting over a work week, which allows for accurate data on physical behaviors and their temporal patterns. Furthermore, we used CoDA procedures to account for the interdependence between different physical behaviors (Dumuid et al. 2018; Gupta et al. 2020b; von Rosen 2023). Another strength is the inclusion of workers in homecare as well as nursing homes from 3 different municipalities of varying sizes, thus providing geographical diversity and increasing generalizability. However, the study also has limitations. While time spent on feet and in sitting were directly measured, the duration of worktime was self-reported by workers in the diary, which could also have introduced some error. Also, data were not collected simultaneously in the 3 municipalities, which may have introduced some bias. We invited all workers in participating eldercare organizations, but workers declining participation may have differed systematically from those who participated in terms of temporal patterns in physical behaviors and/or perceived job demands and resources. Additionally, the proportions of assistant nurses and nurse’s aides as well as of men and women are not perfectly representative of the eldercare workforce in Sweden (Swedish National Board of Social Affairs and Health 2025) which further limits generalizability. Finally, as we lack detailed information on the specific tasks performed, the reasons behind differences between settings remain speculative, highlighting a need for future research on this issue.

## Conclusion

Both homecare and nursing home workers spent most of their working time on feet, but the temporal patterns of time on feet and sitting differed between settings, with homecare workers spending less time in long bouts of sitting relative to moderate and short bouts. Insufficient continuous sitting time may represent a risk for reduced recovery during work. Higher age was associated with more total time on feet, less time in long sitting bouts relative to shorter ones, and less time in moderate bouts of standing compared to short bouts. These results suggest that both the care setting and worker age should be considered when designing workplace interventions aimed at promoting a healthier balance between time on feet and sitting. Nevertheless, while many associations between worker characteristics and physical behavior patterns were statistically significant, most were of small-to-moderate magnitude. As such, the practical relevance of these differences in day-to-day work remains uncertain. Further research is needed to assess whether these behavioral patterns meaningfully impact recovery, health, and long-term work sustainability.

## Supplementary material

Supplementary material is available at *Annals of Work Exposures and Health* online.

wxaf049_suppl_Supplementary_Material

## Data Availability

The data used are available on request.
