# Supplementary material for: Differences in the temporal patterns of occupational time on feet and sitting between homecare and nursing home workers
Source: Ann Work Expo Health. 2025 Aug 29;69(8):855–67. doi: 10.1093/annweh/wxaf049 (PMC12463557; doi:10.1093/annweh/wxaf049)
Supplement: wxaf049_suppl_Supplementary_Material [file wxaf049_suppl_supplementary_material.pdf]

Title: Differences in the temporal patterns of occupational time on feet and sitting between homecare and nursing home workers

Nestor Lögdal[1], Svend Erik Mathiassen[1], Jennie A Jackson[1], David M Hallman[1]

[1]Department of Occupational Health, Psychology, and Sports Sciences, University of Gavle, Gävle, Sweden

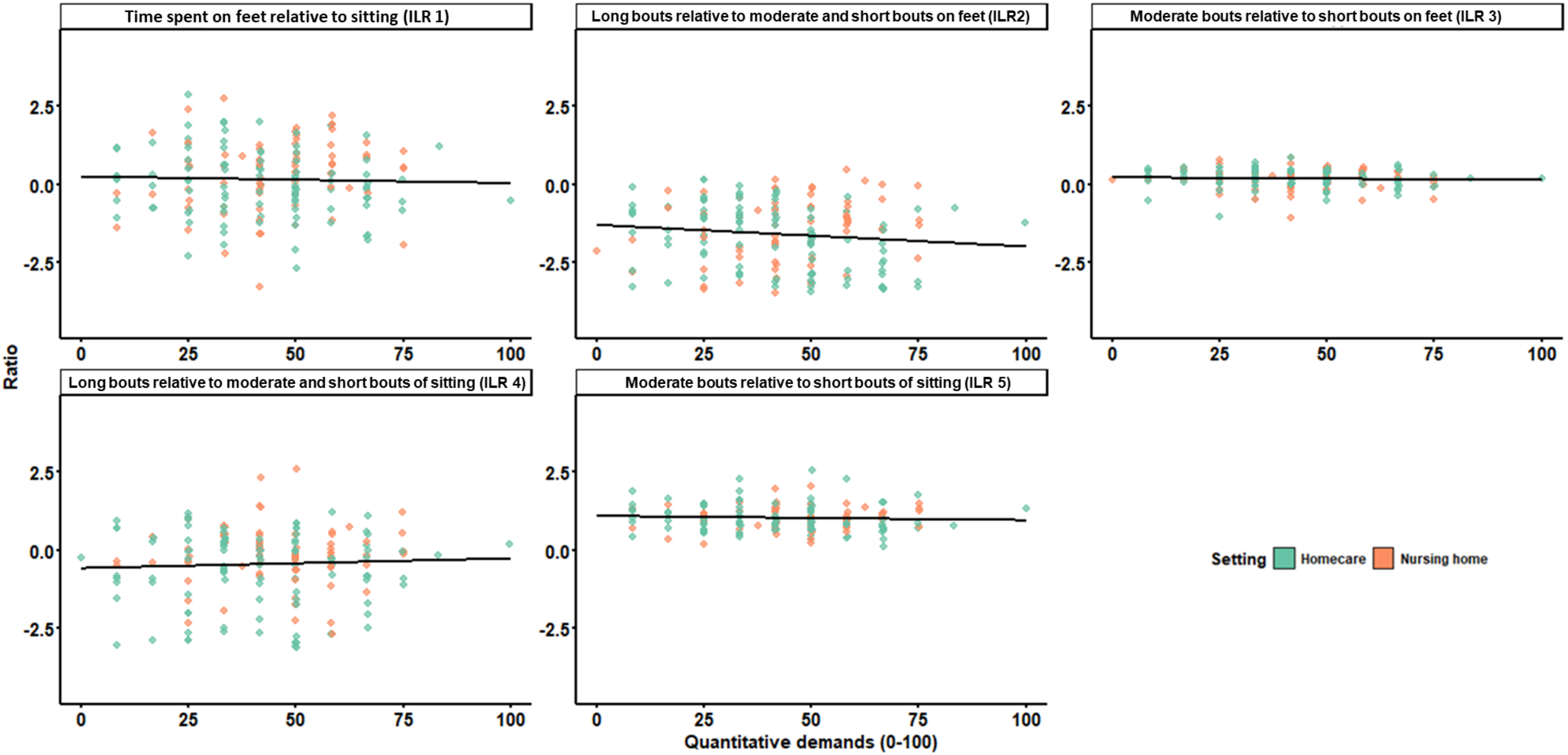

Time spent on feet relative to sitting (ILR 1)
Long bouts relative to moderate and short bouts on feet (ILR2)
Moderate bouts relative to short bouts on feet (ILR 3)
Long bouts relative to moderate and short bouts of sitting (ILR 4)
Moderate bouts relative to short bouts of sitting (ILR 5)
Ratio
2.5
0.0
-2.5
0
25
50
75
100
Quantitative demands (0-100)
Setting
Homecare
Nursing home

Supplementary figure 1. Association between Quantitative demands and physical behaviors in homecare and nursing homes.

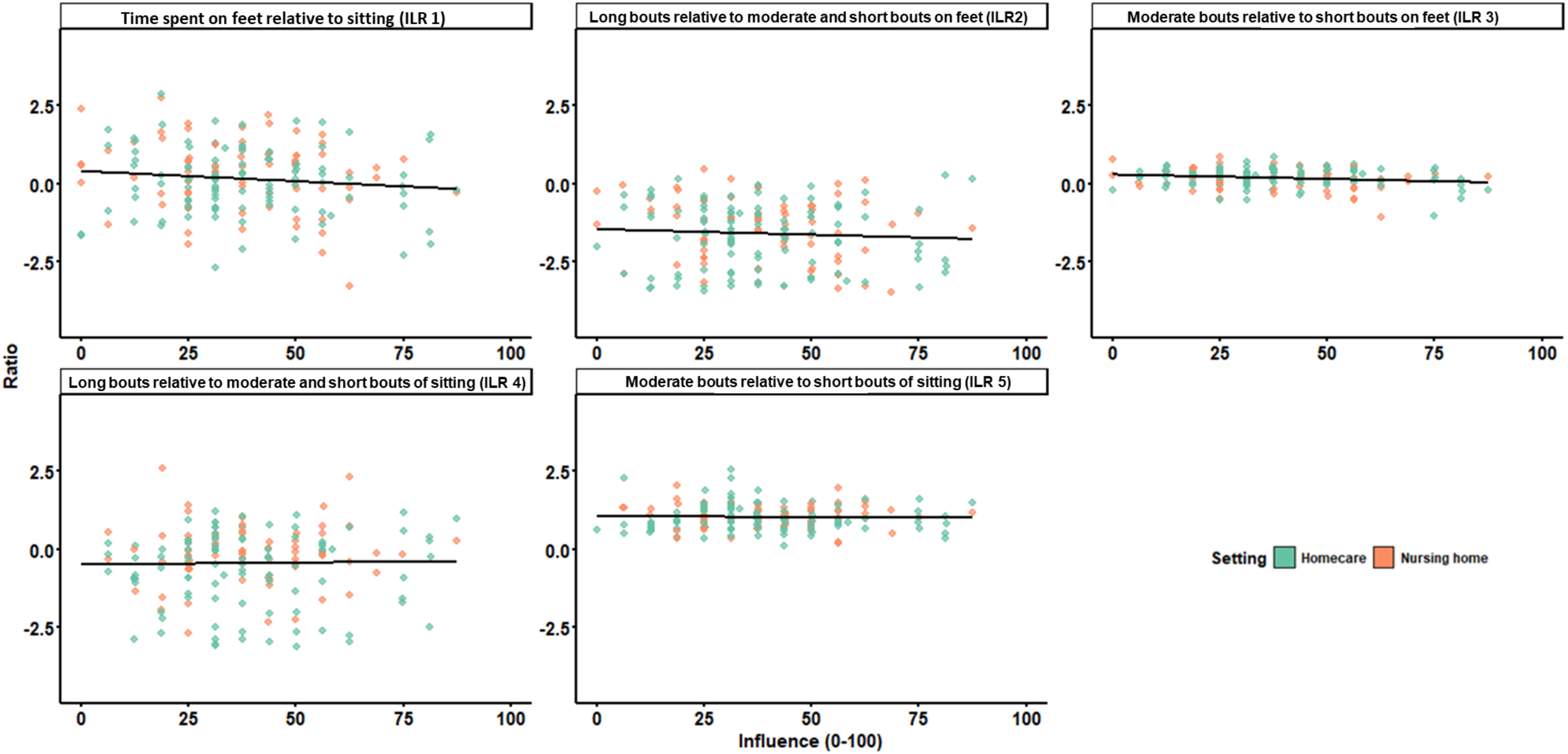

Supplementary figure 2. Association between Influence at work and physical behaviors in homecare and nursing homes.

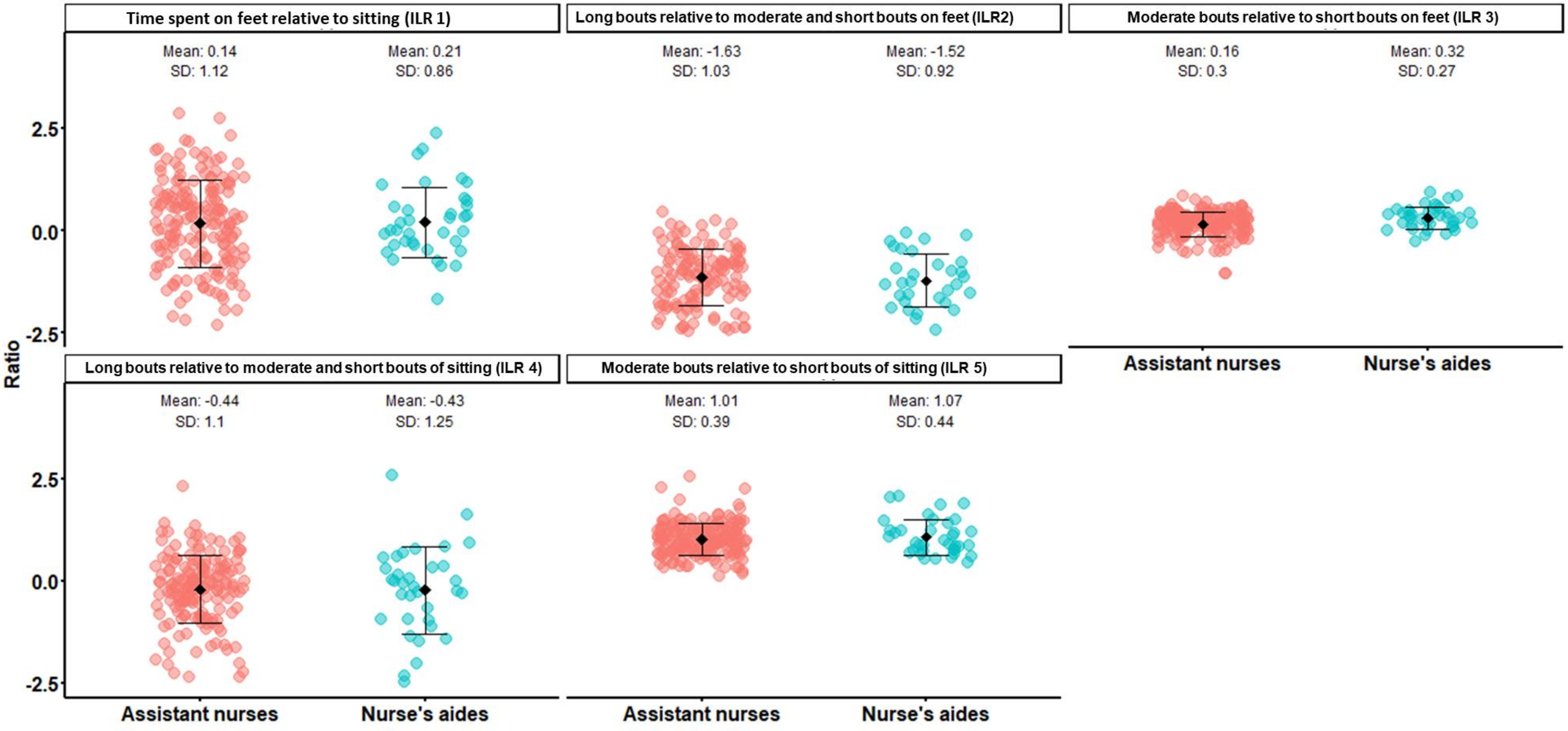
Time spent on feet relative to sitting (ILR 1)
Mean: 0.14
SD: 1.12
Mean: 0.21
SD: 0.86
Long bouts relative to moderate and short bouts on feet (ILR2)
Mean: -1.63
SD: 1.03
Mean: -1.52
SD: 0.92
Moderate bouts relative to short bouts on feet (ILR 3)
Mean: 0.16
SD: 0.3
Mean: 0.32
SD: 0.27
Long bouts relative to moderate and short bouts of sitting (ILR 4)
Mean: -0.44
SD: 1.1
Mean: -0.43
SD: 1.25
Moderate bouts relative to short bouts of sitting (ILR 5)
Mean: 1.01
SD: 0.39
Mean: 1.07
SD: 0.44
Ratio
2.5
0.0
-2.5
Assistant nurses
Nurse's aides

Supplementary figure 3. Association between job title and physical behaviors for assistant nurses and nurse's aides.

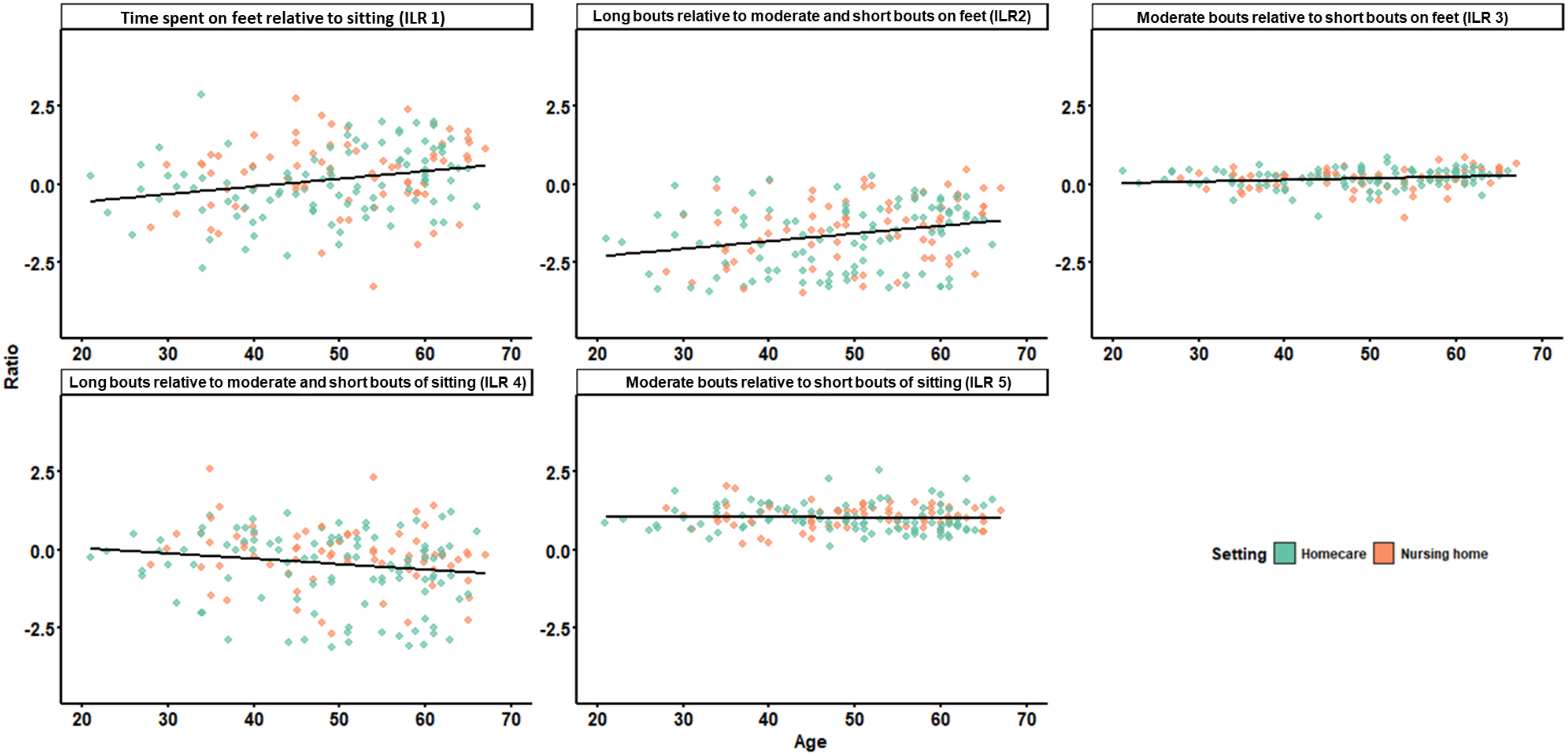

Supplementary figure 4. Association between age and physical behaviors in homecare and nursing homes.

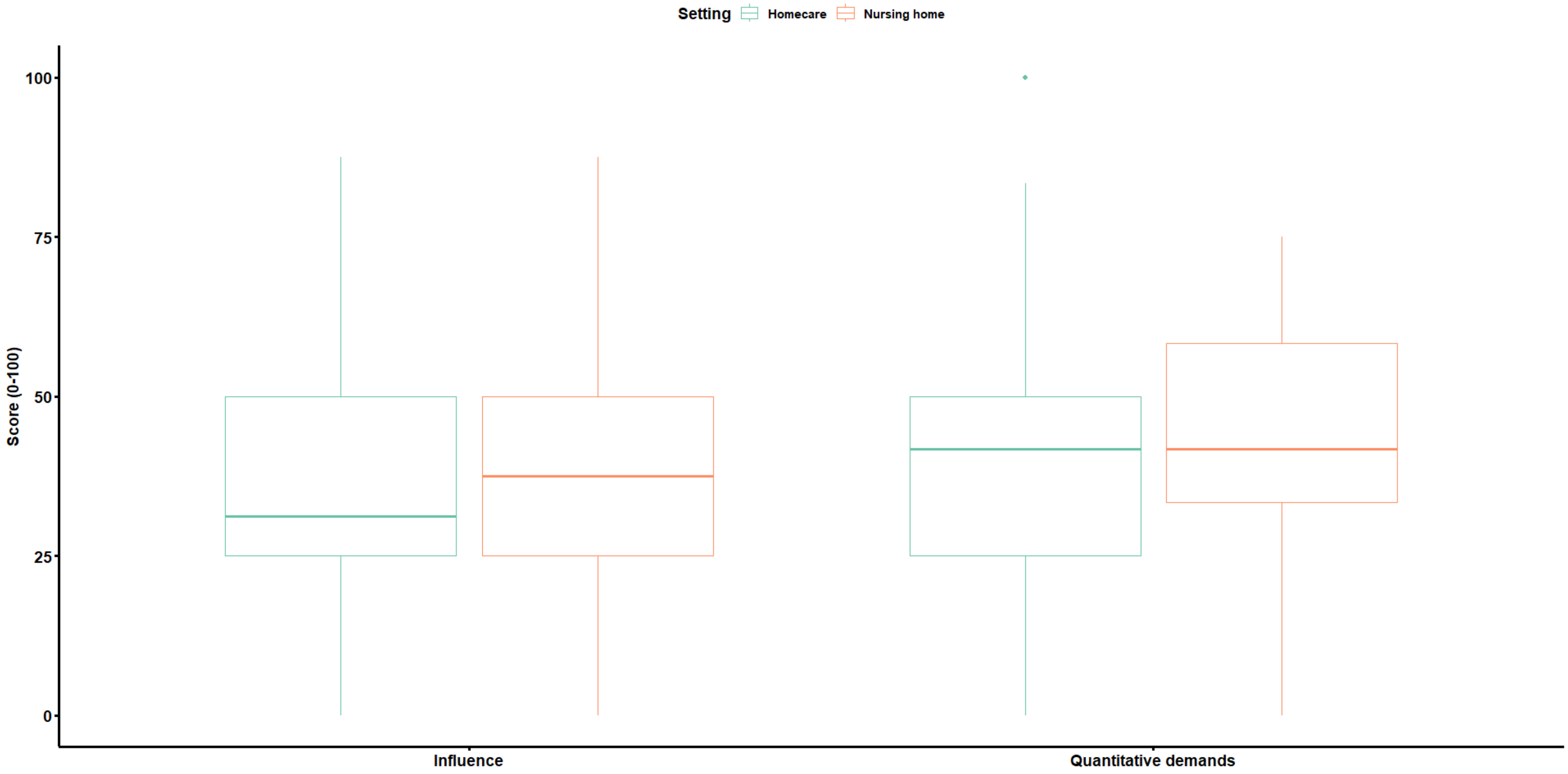

Supplementary figure 5. Boxplots showing the distribution of Influence and Quantitative demands for homecare (green) and nursing home (orange) workers.
